# Atypical painless vision loss in a patient with granulomatosis with polyangiitis

**DOI:** 10.3205/oc000173

**Published:** 2020-12-15

**Authors:** Patricia C. Nelson, Vamsi Kunam, Claudia Prospero Ponce

**Affiliations:** 1 Ophthalmology, Department of Surgery, Texas Tech University Health Sciences Center El Paso, United States; 2 Interventional Radiology, Department of Radiology, Texas Tech University Health Sciences Center El Paso, United States

## Abstract

Vasculitis is a common cause of vision loss, and typically painful. In giant cell arteritis, the most common primary vasculitis in adults, we see elevated inflammatory markers, granulomatous inflammation, and associated headache or scalp tenderness. Vision loss caused by granulomatous with polyangiitis (GPA) is rare and typically associated with pain and orbital findings. Our patient presented for shortness of breath and painless vision loss without orbital inflammation or neural enhancement and a normal fundus exam, suggesting posterior ischemic optic neuropathy. Collaboration amongst sub-specialties and obtaining tissue samples are key to diagnosing granulomatosis with polyangiitis to ensure timely treatment of this fatal and blinding disease.

## Introduction

Granulomatosis with polyangiitis (GPA) is a necrotizing granulomatous vasculitis affecting the small- and medium-sized vessels in the orbit, sinus, lungs, and kidneys [1], [2]. It is a rare disease with the annual incidence being 2.4–11 cases per million, equally affecting both genders of individuals in their 40s–70s [1], [3], [4], [5], [6]. Ocular findings are seen in 15–60% of cases and can be some of the earliest findings [1], [2], [3], [5], [7]. Nearly 50% of patients can develop painful scleritis, as well as other ocular signs and symptoms including uveitis, keratitis, and orbital inflammation resulting in proptosis, diplopia, and epiphora. If orbital findings are present, 20–50% of patients can develop severe vision loss [1], [3], [8], [9]. Painless vision loss without orbital findings has rarely been reported [1], [10]. High clinical suspicion and collaboration with multiple sub-specialties is required to reduce mortality and morbidity.

## Case description

### History

A 64-year-old Caucasian woman presented to the ER for shortness of breath following a recent sinus surgery for a deviated septum. During her stay, ophthalmology was consulted for right vision loss. The patient was being treated for cavitary pneumonia with suspected invasive aspergillosis. She denied any eye pain, headache, scalp tenderness, or jaw claudication, and could not remember the date of onset of her vision decline, except that it was following recent sinus surgery.

### Examination

On clinical exam, her vision was no-light-perception (NLP) in the right eye with a relative afferent pupillary defect, and 20/20 vision in her left eye. Anterior and posterior exam was normal in both eyes with no evidence of proptosis, optic nerve edema, hemorrhages, cotton wool spots, or pallor. Initial work-up showed leukocytosis (34.15), elevated erythrocyte sedimentation rate (90), and C-reactive protein (3.51). CT chest showed cavitary lesions, and a bronchio-alveolar lavage showed increased galactomannan, leading to a diagnosis of suspected aspergillosis. A magnetic resonance imaging (MRI) of the brain and orbits showed no optic nerve abnormalities, enhancement, or orbital inflammation. The patient received amphotericin B and IV steroids with symptomatic improvement except for her vision. The patient declined a lung biopsy. Low-dose oral steroids, 40 mg prednisone, were continued and not increased due to suspected aspergillosis pneumonia.

### Clinical course

**Figure 1:**
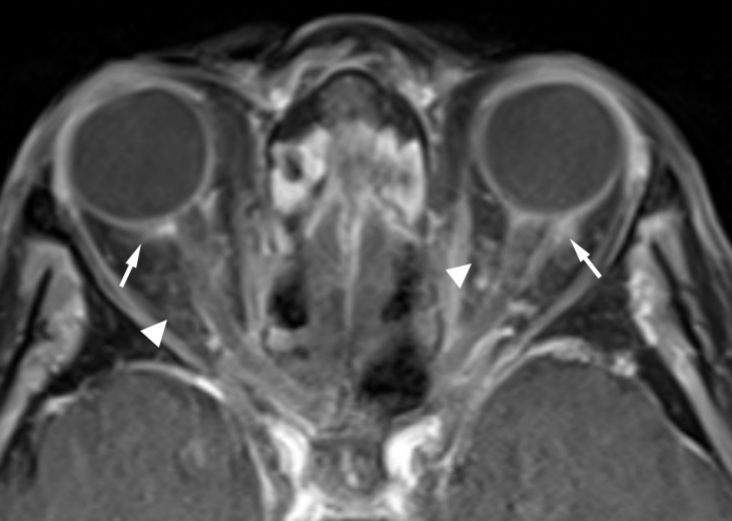
Post-Gd axial T1-weighted MR image with fat saturation shows enhancement of bilateral posterior sclera extending to the adjacent optic nerve sheath (arrows); findings are consistent with scleritis. Also noted is mild ill-defined enhancement in retro-conal fat (arrowheads), consistent with mild post-septal cellulitis.

Three weeks later, oral prednisone had been discontinued and the patient began developing new ocular symptoms in her left eye as well as a right 3^rd^ nerve palsy. C-ANCA positivity was confirmed. Despite high-dose steroids, the patient progressed to NLP vision in the contralateral left eye, and repeated MRI demonstrated interval development of acute left optic nerve ischemia and bilateral intra-orbital inflammation (Figure 1 (Fig. 1)). Repeat ENT biopsy was obtained and demonstrated focal arteritis with some giant cells and no invasive fungal elements, ruling out invasive aspergillosis and confirming the suspicion of GPA. The patient developed a nasal deformity with a saddle nose, cotton wool spots in the left retina, and acute renal failure requiring dialysis. Reinstitution of high-dose steroids and adjunctive cyclophosphamide reversed the patient’s acute renal failure and the 3^rd^ nerve palsy, but did not reverse her vision loss.

## Discussion

Granulomatosis with polyangiitis is a rare disease and often affects multiple organ systems. Ear, nose and throat (ENT) signs can be present in 70–100%, lung involvement in 50–90%, and ocular involvement in 15–60% of cases [1], [3], [4], [5], [7]. Severe vision loss or total blindness can be seen in 8–37% of cases [3], [11]. Collaboration between subspecialties is critical in these rare conditions – remembering Occam’s razor, we must look for a diagnosis that explains all multi-organ symptoms. Nasal tissue biopsy ruled out aspergillosis and suggested changes of vasculitis with diffuse necrosis. Pathologic tissue confirmation is highly recommended in patients when imaging and other laboratory studies are insufficient.

## Conclusions

1. Granulomatosis with polyangiitis can present with painless vision loss without other orbital or ocular findings.
2. A multidisciplinary approach is essential for timely diagnosis and management of granulomatosis with polyangiitis.
3. Tissue specimen is essential to diagnose GPA and rule out other confounding etiologies.

## Notes

### Competing interests

The authors declare that they have no competing interests.
